# Migration of Avocado Virgin Oil Functional Compounds during Domestic Cooking of Eggplant

**DOI:** 10.3390/foods10081790

**Published:** 2021-08-02

**Authors:** Cristina Samaniego-Sánchez, Sandra Teresita Martín-del-Campo, Ma. Claudia Castañeda-Saucedo, Rosa María Blanca-Herrera, José Javier Quesada-Granados, Jessica del Pilar Ramírez-Anaya

**Affiliations:** 1 Department of Nutrition and Bromatology, Pharmacy Faculty UGR, Campus Cartuja s/n, C.P. 10871 Granada, Spain; csama@ugr.es (C.S.-S.); rblanca@ugr.es (R.M.B.-H.); quesadag@ugr.es (J.J.Q.-G.); 2 Escuela de Ingeniería y Ciencias, Tecnologico de Monterrey, Epigmenio González 500, Querétaro C.P. 76130, Querétaro, Mexico; smartinde@tec.mx; 3 Department of Nature Sciences, Centro Universitario del Sur (UdeG), Av. Enrique Arreola Silva 883, Ciudad Guzmán C.P. 49000, Jalisco, Mexico; claudia.saucedo@cusur.udg.mx; 4 Department of Computational Sciences and Technological Innovation, Centro Universitario del Sur (UdeG), Av. Enrique Arreola Silva 883, Ciudad Guzmán C.P. 49000, Jalisco, Mexico

**Keywords:** Mexican avocado oil, phytosterols, stanols, polyphenols, fatty acids, antioxidant activity, fry, thermal treatment, boil

## Abstract

Avocado virgin oil (AVO) was used during eggplant deep-frying, boil, and boil in a water-oil mixture (W/O). There were measured the contents of moisture, dry matter, fat, total (TPC) and ten individual phenols, antioxidant activity (ABTS and DPPH), and total sterols; as well as the profiles of eight fatty acids and fourteen sterols/stanols. The values of raw and processed foods were compared and studied with multivariate analysis. The antioxidant capacity of AVO lowered after deep frying but augmented in eggplant and water after all treatments. The TPC was steady in AVO and raised in fried eggplant. Thermal treatments added to the initial profiles of the AVO, eggplant and water, nine, eight, and four phenols, respectively. Percentages of the main fatty acids (oleic, palmitic and linoleic), and sterols (β-sitosterol, campesterol, and Δ5-avenasterol), remained unchanged between the raw and treated AVO; and the lipidic fractions from processed eggplant. Cooking leads to the movement of hydrophilic and lipophilic functional compounds between AVO, eggplant and water. Migration of sterols and unsaturated fatty acids from AVO to eggplant during deep frying and W/O boiling improved the functional properties of eggplant by adding the high biological value lipophilic fraction to the naturally occurring polyphenols.

## 1. Introduction

Avocado (*Persea americana*) is produced worldwide, but México is recognized as the leading producer with more than 32% of the harvested volume FAO [1]. Although avocado is mainly consumed in its fresh form, the production of avocado oil is a growing industry [2]. Avocado oil is a functional oil extracted from the pulp fruit, is recognized from its chemical properties; as the fatty acid profile with a high proportion of unsaturated fatty acids (oleic and linolenic) and a small proportion of saturated acids; and the presence of bioactive phytochemicals such as phytosterols, tocols, carotenoids and polyphenols [3,4,5,6]. Phytosterols in AO have been measured in higher concentrations than sunflower, palm and virgin olive oils [7]. On the other side, eggplant (*Solanum melongena* L.) is an important source of phytochemicals in countries of the Mediterranean region [8]; it is recognized for its high anthocyanin content [9], polyphenol richness, and its associated high antioxidant activity [10]. Phytochemicals in virgin oils and vegetables have received increased interest from consumers and researchers for their beneficial health effects. The health benefits associated with those compounds include a wide range of effects mostly studied on cellular or animal models such as antioxidant activity, anti-inflammatory properties on chondrocyte cells, prevention of cardiovascular disease, lowering of low-density lipoprotein-cholesterol and triglyceride levels, reduction of the inflammatory events and positive results in biochemical indicators of metabolic syndrome, inhibition of prostate cancer cell growth, enhancement of the absorption of A, E, and K vitamins, chlorophylls, acetogenins and carotenes [5,11,12,13,14,15].

Cooking is a necessary step before the consumption of many vegetables. It allows the improvement of sensory, nutritious and safety features; and modifies the phytochemical digestibility. Cooking methods can change phytochemical profiles positively or negatively [16,17]. It is known that temperature, time, area exposed to air, number of frying sessions, vegetable, and oil processed, are factors influencing the extent of changes into the oil and vegetable [18,19,20,21,22]. In eggplant has been studied the effect of various processing techniques in the phenolic compounds present. In cooking dry methods: frying, grilling, and baking increased total phenolics, flavonols, and antioxidant activity of eggplant [8,23,24,25]. it is known that it retains a high amount of oil during cooking due to the high affinity of the carbohydrate structure to oil [26]. On the other hand, whet cooking methods (boiling and pressure cooking) increase polyphenol extractability and antioxidant capacity (AC).

The effect of processing in oil depends on the compound analyzed and the cooking technique assayed. Polyphenolic compounds in sunflower, soybean, canola and olive oils are better preserved during low-fat frying with microwave grilling and oven than deep-frying [27]; a net decrease of polyphenol species was measured in extra virgin olive oil after boil, boil in a water-oil mixture, deep fry, and sauté, of tomatoes, eggplant, potato and pumpkin [28,29]. The effect in sterol content of oil has been studied in long term treatments at high temperatures; after prolonged heating (48 h, 170 °C) of rapeseed oil was measured a steady decrease in sterol content ranging from 21% to 46% depending on the kind of oil analyzed (virgin or refined) [19]; it was also found in soybean, and in olive oil heated during 30 to 120 min, the reduction was more intense at 180 °C than at 120 to 150 °C [30]. Repeated frying sessions reduce sterol content in sunflower, palm oil, cottonseed oil, and virgin olive oil [7].

Oil not only acts as an effective medium for energy transfer from the heat source to the food, it also adds flavor, nutritional compounds, and interacts with vegetables through the migration of functional compounds in both directions [18]. There has been demonstrated polyphenol movement between oil (olive oil) and the vegetables included in sofrito preparation [31]. Ramírez–Anaya et al. [10] stated that vegetables processed in virgin olive oil develop specific phenolic and antioxidant activity profiles resulting from the characteristics of the raw vegetables and the cooking technique used, and also migration between both matrices [28]. The movement of sterol is done from rich sources (oils) to processed vegetables. At the end of frying, an increase in sterol content of potatoes from oils such as sunflower, palm, cottonseed or virgin olive oil was reported. The content in potato was the same as that measured in the different oils assayed [7].

Avocado oil is mainly consumed cold into salads as a seasoning, but the use as cooking fat as many other virgin or extra virgin oils has been scarcely studied [32]. Oil stability is affected by the initial avocado oil composition. It may be affected by processing factors, but most studies focus on the characteristics of fresh avocado oil and the impact of geographical origin, genetic diversity, physiological stage, and extraction technologies in its composition [33,34,35,36,37,38]. The present research aims to study the effect of different domestic cooking techniques on the qualitative and quantitative composition of phenolic compounds, sterols, and fatty acids of Mexican avocado virgin oil and eggplant.

## 2. Materials and Methods

### 2.1. Standards and Reagents

The standards were provided as follows: vanillic acid, syringic acid, gallic acid, pinoresinol, p-coumaric, caffeic acid, o-coumaric and ferulic acids, chlorogenic acid, tyrosol, quercetin, apigenin, luteolin, p-hydroxybenzoic and p-hydroxyphenylacetic acid, Trolox ((±)-6-hydroxy-2,5,7,8-tetramethyl-chroman-2-carboxylic acid), and fatty acids methyl ester (Supelco 35 Component FAME reference standard mixture) from C4 to C24 were obtained from Sigma-Aldrich SL (Madrid, Spain). α-cholestan (purity ≥ 95%) standard from Sigma-Aldrich (St. Louis, MO, USA). Tyrosol and o-vanillin standards were supplied by Fluka Chemicals (Madrid, Spain). Rutin by HWI Analytik GMBH pharma solutions (Frankfurt, Germany), and oleuropein by Extrasynthèse (Genay, France). Hydroxytyrosol standard was synthesized at the Organic Chemistry Laboratory (Faculty of Pharmacy of Granada University).

All reagents used were analytical grade. ABTS (2,2′-azinobis-(3-ethylbenzothiazoline)-6-sulphonic acid), DPPH (2,2-diphenil1-picryl hydrazyl), methanol, ethanol, sodium chloride, anhydrous diethyl ether, potassium hydroxide, sodium sulfate, hexane, and all reagents for fatty acid profile and sterol compounds were supplied by Sigma-Aldrich SL (Madrid, Spain). Potassium peroxodisulfate (K_2_S_2_O_8_) by Panreac Quimica SL (Barcelona, Spain); Folin-Ciocalteu’s phenol reagent was from Merck (Darmstadt, Hesse, Germany), and anhydrous sodium carbonate (Na_2_CO_3_) from Carlo-Erba (Rodano, Milan, Italy). HPLC water was obtained from A Milli-Q purification system (Millipore, Bedford, MA, USA).

### 2.2. Foodstuffs and Cooking Conditions

Five liters of Mexican virgin avocado oil (VAO) in one-liter glass bottles were proportionated by Aceitera Mevi México Company located at Zapotiltic, Jalisco, Mexico. The oil was bottled in amber glass containers (500 mL) with nitrogen in the headspace and stored at 4 °C. Fresh eggplants (*Solanum melongena*) were purchased in Granada (Spain) at commercial establishments. Three vegetable units were washed, peeled off, and cut into cubes (1 cm side). Changes in the VAO, vegetable, and water under four culinary techniques commonly used at the domestic level were assayed: deep-frying, conventional boiling, and boiling in a bilayer mixture of water and VAO (W/O). Uncooked foods were included as a control treatment. All assays were done in a stainless-steel vessel (16 cm diameter and 1.5 L capacity) to avoid variations from the oxygen exposition. An amount of 120 g of eggplant was used in each assay. To fix the temperatures and the proportions (*w*/*w*) between cooking medium and vegetable were consulted the most frequently used conditions in Spanish and Mexican traditional recipes: Deep-frying was done at 180 °C in a 5:1 (AVO:vegetable) proportion. Boiling was assayed at 100 °C, with a 5:1 (water:vegetable) proportion in the conventional variant, while in W/O boiling, the proportion was held at 4.5:0.5:1 (water:AVO:vegetable). In all cases, the temperatures of the eggplant cubes were maintained for 10 min and then were drained for 5 min. The drained bilayer mixture from W/O boiling was transferred into a decantation funnel for phase separation.

From this point, vegetable, oil, and water were treated separately, proceeding to homogenization once cooled under refrigeration. Each sample was subsequently protected from light and held under cold. Tests were repeated three times.

### 2.3. Determination of Moisture, Fat, and Dry Matter Content

The moisture, fat, and dry matter (DM) contents of the vegetable, EVAO, and water were analyzed using AOAC methods [39]. Results were reported as a percentage (%).

### 2.4. Extraction Conditions of Phenolic Compounds

The extraction of phenolic compounds was conducted using, as solvents, acetone or methanol solutions according to Saura-Calixto [40] for eggplant and Montedoro et al. [41] for VAO. The sample amount used was 2 g for vegetable or 4 g for oil, in a sample:solvent ratio of 2:1 and 1:1, respectively. The boiling water recovered from experiments was directly analyzed after centrifugation to eliminate solid particles under the conditions referred by Tabart et al. [42]. The mixtures were stored as aliquots at −80 °C until analysis.

### 2.5. Individual Phenolic Compounds

The identification of the polyphenols was carried out in a UPLC-QTOF-MS [43] with a modification of the method described by Esteban-Muñoz et al. [44].

#### 2.5.1. Chromatographic and Mass Spectrometer Operating Conditions

The electrospray ionization (ESI)-MS experiments were performed on a liquid chromatography system with a hybrid mass spectrometer SYNAPT G2 HDMS Q-TOF model (Waters, Milford, CT, USA). The UPLC separation was conducted in a C18 ACQUITY UPLC HSS T3 column (2.1 cm × 100 mm internal diameter, 1.8 µm film thickness) (Waters, Milford, CT, USA). The program for chromatography was described by Esteban-Muñoz et al. [44]. Ten microliters of sample were injected, and the flow rate was 0.4 mL/min. TOF conditions (measurement ranged from 50 to 1200 Da with the following mass spectrometer settings: nebulizer gas: 2 bar; capillary: 3100 V; drying temperature: 180 °C; drying gas: 6 L/min) consisted of a full MS, and data-dependent scanning was performed in negative mode with electrospray ionization (ESI). The acquisition of fragment spectra for each mass was carried out using collision energy set to 40 eV with MS/MS mode.

#### 2.5.2. Identification and Quantification

Phenolic compounds were identified using the exact mass, isotopes by comparing negative masses from previously recorded research and comparison of detected fragments ions with databases (ChemSpider, FooDB, and PubChem) using MassLynx v4 software (Waters, Milford, CT, USA) for instrument control, data acquisition, and data analysis. We carried out a calibration curve with a good R2 to ensure linearity for each phenolic compound selected. The standards were analyzed under the same working conditions as the samples.

### 2.6. Determination of Antioxidant Capacity

The antioxidant capacity was measured with DPPH and ABTS methods [45,46], slightly modified and adapted to microscale (20 µL of oil/water/vegetable extract was used in both antioxidant methods) [28,47,48]. The absorbance was measured at 515 and 734, respectively, using iNanoQuant (Tecan) Infinite M200 Pro Multimode Microplate (Männedorf, Switzerland) and Software program i-control v 1.8 for the infinite rider (SelectScience, Bath, UK). These methods used Trolox as the external standard. The expression of results was as micromoles of Trolox equivalents per gram of fresh sample (µmol of TE/g f.w.). CA values are the mean ± standard deviation of three experimental replicates.

### 2.7. Total Phenolic Content (TPC)

The TPC was determined employing the Folin–Ciocalteu method [49] with some modifications [28]. The blue color formed after standing the samples, and the blank at ambient temperature was measured at 750 nm (BOECO S-22 UV-VIS spectrophotometer, Hamburg, Germany). The phenolic contents were calculated by interpolation from a calibration curve of gallic acid standard dissolved in methanol (0.05–0.70 mg/mL) (r = 0.999). Results were expressed as milligrams of gallic acid equivalents per kilogram of fresh sample (mg GAE/kg f.w.).

### 2.8. Evaluation of Lipidic Fraction

#### 2.8.1. Extraction

To obtain the lipid fraction from processed eggplant was used the procedure of Prandini et al. [50] based on a modification of the Folch method [51]. An amount of sample was combined (10:1) with a chloroform:methanol mixture (2:1) in an Ultraturax device. The mixture was homogenized by continuous agitation for 30 min and then centrifuged (8000 rpm, 15 min). Once recovered, the supernatant was added with sodium chloride solution and mixed to repeat the agitation and centrifugation. The organic phase was evaporated in a rotary evaporator at room temperature, and the residue was re-dissolved in 3 mL hexane in amber vials. The sample was stored at −80 °C until preparation and analysis of fatty acid methyl esters (FAME) and sterols.

#### 2.8.2. Derivatization of Fatty Acids for GC-MS Analysis

A modified method described by Christie [52] and used in our laboratory [53] was followed for obtaining fatty acid methyl esters (FAME).

#### 2.8.3. Chromatographic and Mass Operating Conditions

Gas chromatography was performed on a QUATTRO micro GC system equipped with a split/splitless injector, and a flame ionization detector (FID) with ionization mode E+ connected to a mass spectrometer with tandem quadrupole; Using a capillary column (length: 30.0, internal diameter: 250 µm) (Waters, Milford, MA, USA). All the chromatographic and mass operating conditions (inlet, detector and MS detector temperatures, injection volume, split ratio, oven program, and split ratio) were fixed according to Sánchez-Hernández et al. [53]. The software used for data processing was MassLynx V4.1 (Waters Inc., 2010, Milford, MA, USA). The fatty acids were identified relative to retention times of FAME mix external standard, followed by area normalization for the calculation of percentages of fatty acids.

#### 2.8.4. Preparation and GC-FID Analysis of Sterols Compounds

The sterol identification and quantification were determined according to the official European analysis methods [54,55]. The compounds were extracted and silylated previously to the chromatographic separation and analysis on a GC–FID Varian 450 (Torino, Italy), with a Phenomenex capillary column (30 m × 0.25 mm internal diameter × 0.25 µL film thickness) (Torrance, CA, USA). The oven temperature was programmed from 250 °C to 280 °C, using helium (Gasin, Porto, Portugal) as the carrier gas. Software SW ChemStation vD.01.00 (Santa Clara, EEUU) was used for data acquisition. Total sterol content was expressed in mg/kg, and sterol profile as a percentage (%).

### 2.9. Statistical Analysis

Statistical analysis was performed using the Statistica v13 software (TIBCO Software Inc. (2017), Tulsa, OK, USA). The obtained data were separated into two datasets, the first dataset for the compositional parameters and the activities and compounds from the hydrophilic fraction; the second dataset corresponded to the lipophilic fraction. First, analysis of variance (ANOVA) was applied to each dataset to identify the parameters showing significant differences (*p* < 0.05) according to the cooking technology and the foodstuff. In addition, the Fisher’s least significant difference (LSD) test was applied to each significant factor. LSD is a post-hoc test that compares the difference between a couple of means grouping them. The means without common letters present significant statistical differences (*p* < 0.05) calculated using the error means square of the corresponding ANOVA.

Finally, general discriminant analysis (GDA) was applied for each dataset separately and the whole original group of data to discriminate samples according to the cooking technology. For GDA, the forward stepwise analysis was performed (*p* to enter = 0.05, *p* to remove = 0.05) to reduce the number of initial variables by selecting a subset of uncorrelated variables.

## 3. Results

### 3.1. Composition of Raw Foods

#### 3.1.1. Hydrophilic Fraction

##### Fat and Moisture Content

Fat content reached 0.02% in raw eggplant (Table 1), and was null in water. The amount of moisture content in the oil, vegetable, and water reached 0.1, 93.3 and 99.0%, respectively, while the dry matter in the same foods was 0, 6.7, and 0.1%, respectively. Values measured in raw water were omitted in tables because of null values.

##### Antioxidant Capacity

In raw eggplant and oil, the magnitude of the antioxidant capacity measured by the ABTS method was higher than that of DPPH (Table 1).

In raw AVO, values of ABTS antioxidant capacity reached 1.7 mM TE/kg (Table 1). This value coincides with that of AO extracted using ultrasound-assisted aqueous extraction (1.7 mM TE/kg) but was lower than AO extracted by supercritical CO_2_ (4.5 mM TE/kg) and by solvents (2.9 mM TE/kg) [4]. Oil values of lipophilic antioxidant activity in AO have been described as weak, similar to those reported for flaxseed oil extracted with solvents [36,56]. Some authors [4,57] reported a positive correlation between AO sterols and ABTS and DPPH antioxidant capacities.

In raw eggplant, our results of antioxidant activity measured by DPPH and ABTS (Table 1) were superior to those values previously reported in eggplant (0.6 and 1.3 μmol TE/g f.w.) [10]. However, it ranges in the same order. Eggplant have a recognized high antioxidant capacity varying according to eggplant variety, fruit shape, and size [9]. In this vegetable, the antioxidant capacity and phenolic content are highly positively correlated [58].

##### Total Phenolic Content

As can be seen in Table 1, the magnitude of TPC found in raw AVO (8.9 mg GAE/kg) agreed with previous studies; such as those of 5.1 to 11.8 mg catechinE/g of Mexican Hass AO [36], or from 0.2 to 4.4 mg/100 g measured in oil extracted after microwave drying pretreatment from unripe or ripe unpeeled fruits [3].

Our TPC in raw eggplant were 35.3 mg GAE/kg f.w. Previously were reported values of 65.5 mg GAE/100 g [24], 22 mg GAE/100 g f.w. in eggplant from India [59]; 102.9 to 400.8 mg ChlorogenicAE/100 g f.w. in Spanish accessions [60], and 13.5 mg GAE/100 g d.w. in Japanese samples [25].

##### Phenolic Profile

Into raw AVO were measured tyrosol, p-vanillin, vanillic acid, ferulic acid, apigenin, quercetin, chlorogenic acid, and tyrosol. Tyrosol has not been previously reported in AVO. Previous studies in raw AVO extracted after microwave drying pretreatment also referred to vanillic acid, ferulic acid, quercetin, and others as coumaric gallic 3,4-dihydroxyphenylacetic p-hydroxybenzoic acids were not found in our study [3].

The diversity of polyphenolic compounds present in raw eggplant is widely recognized, but in our study, only were confirmed with standards chlorogenic acid, tyrosol and p-vanillin, being chlorogenic the most abundant (37.8 mg GAE/kg f.w.). Compounds found in our study have been previously reported in raw eggplant, additionally to caffeic acid [61], ferulic, and p-coumaric acid [24,62]. Chlorogenic acid is a hydroxycinnamic acid-derivative; this family of phenolics accounts for 95.6% of the total identified phenolic compounds in eggplant [24].

#### 3.1.2. Lipidic Fraction

##### Fatty Acid Profile

In raw AVO, the most abundant fatty acid, in order of decreasing amount (Table 2) are, oleic (65.3%), palmitic (14.4%), and linoleic (9.1%). These three FAs make up around 88.7% of all FA profiles. The next most abundant FAs are palmitoleic (6.4%) and cis-vaccenic (4.0%), which account for a further 10.4% of FAs. Also were measured in little amounts of gondoic (0.3%), linolenic (0.4%), and dihomo-γ-linolenic (0.2%) acids. The only saturated FA was palmitic, its proportion small compared with the sum of monounsaturated (76.0%) and polyunsaturated (9.6%) FA percentages. The fatty acid profile varies slightly from those published in the literature for Hass avocado oils obtained through centrifugation, or with the aid of microwave pretreatment, or sono-physical process [34,35,37]; but contrary to these studies, stearic acid was not found in our experimental raw oil [34].

##### Total and Individual Sterol Stanol Contents

Total sterol content in raw AVO attained 3881 mg/kg. β-sitosterol is the main sterol, with 85.1% of the total sterol content (TSC), followed at much lower percentages by campesterol (5.9%), 5-avenasterol (5.1%), and clerosterol (1.5%). Stanols accounted in a very small proportion (1.0%) to the TSC, being the main compounds sitostanol (0.4%), and Δ5,23- and Δ5,24-stigmastadienol (0.3 and 0.2%, respectively). In an almost negligible percentage were quantified campestanol and Δ7-stigmastenol.

The TSC in VAO was in the range found in 22 commercial avocado oils (2859 to 5955 mg/kg of oil) of American and Spanish origin [63].

Sterol profile differed from that of rapeseed oil in such a way that β-sitosterol, campesterol, and avenasterol are also predominant, but its proportions (39.8, 29.6, 17.3%, respectively) are quite different from that found by us in AVO [19]. It is the same as virgin olive oil (48.6, 6.2 and 17.8%), but the proportions are similar to that of cottonseed oil (86.8, 8.8 and 2.6%). Otherwise, our data of sterol profile was completely in agreement with profiles measured in 22 commercial avocado oils; the authors [6] referred to β-sitosterol as predominant, followed by 5-avenasterol, campesterol, and stigmasterol in minimum quantities. We found stigmasterol in very low percentages, being surpassed by clerosterol. Based on previous studies, it was assumed that raw eggplant has a negligible TSC. It has been reported that the raw vegetable had the lowest TSC (29 mg/kg) after wax gourd among 33 foods studied [64].

### 3.2. Changes in Foods after Cooking

#### 3.2.1. Hydrophilic Fraction

##### Fat and Moisture Content

Eggplant showed significant variations in fat content (Table 1) between the raw (0.02%), deep-fried (37.8%), and boiled W/O (6.4%) vegetable (*p* ≤ 0.05); but raw and boiled cubes did not differentiate according to this variable (*p* ≥ 0.05).

Moisture content in oil and water was significantly steady after cooking (*p* ≥ 0.05) (Table 1). It was nearly null (0.1%) in oil, but it reached 99.9% in water. On the other side, fresh eggplant significantly reduced its moisture content after being processed by deep-frying (45.9%) and W/O boiling (89.5%) (*p* ≤ 0.05); but increased 3% from the initial moisture content after boiling (*p* ≤ 0.05).

AVO did not gain in dry matter, remaining null after cooking eggplant (Table 1). However, uncooked water did attain 0.5% after boiling and 0.6% after W/O boiling (*p* ≤ 0.05). The dry matter of the eggplant changed according to processing treatment (*p* ≤ 0.05), diminishing with boiling (3.5%); but increasing when AVO was included in cooking. The reduction resulted from lixiviation and solubilization phenomena; however, the increment comes from the simultaneous gain in fat and loss of moisture during deep-frying (54.1%) and W/O boiling (11.8%).

It has been documented a strong relation between moisture loss from fried foods and oil absorption. Normally, water evaporation is rapid during frying, and the free space is filled with the absorbed oil [18]. Nevertheless, adhesion of oil at the outer edges of the eggplant surface may explain the increment in fat content seen during W/O boiling, as previously demonstrated in a study where was found a high gain of oil in eggplant during cooling and lifting off from cooking recipient [65]. Additionally, the eggplant pulp has such a high affinity to fats, in a way that has been studied as a potential natural emulsifier [26].

##### Antioxidant Capacity

In the eggplant, oil, and water recovered from culinary assays, the magnitude of the antioxidant capacity measured by the ABTS method was higher than DPPH values, but the tendencies of the results were similar in both cases (Table 1). The antioxidant capacity measured by DPPH in the oil recovered from experiments did not differentiate between culinary treatments, but the reduction measured by ABTS after deep frying was significant and clear. This property measured in raw oil was not significantly modified after W/O boiling.

Fresh eggplant increased its antioxidant capacity after all of the culinary techniques were assayed. Deep frying was the technique that increased this property in a more accentuated way, followed by the W/O boil and conventional boil.

Water boiled by the two modalities acquired measurable antioxidant capacity (ABTS and DPPH). The results obtained by the ABTS method shown that the addition of AVO during boiling of eggplant more increased the antioxidant capacity in water than did conventional boiling (*p* ≤ 0.05). This behavior agreed with previous observations in cooking water after boiling with or without the addition of extra virgin olive oil [28].

##### Total Phenolic Content

In AVO, the TPC was almost steady wherever the culinary treatment was used. In raw eggplant remained unchanged after the two boiling modalities (*p* ≥ 0.05) but increased in 605% after deep frying (Table 1). The 1196% increase in chlorogenic acid concentrations most explained the rise in TPC in eggplant after cooking.

##### Phenolic Profile

**Figure 1:**
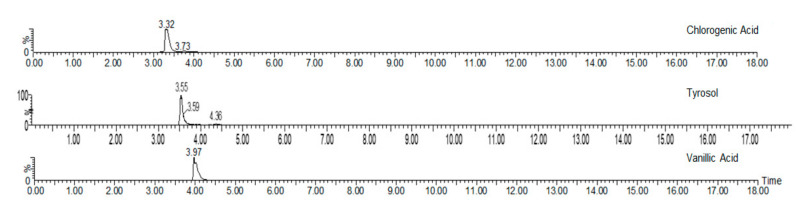
Chromatograms of the most abundant phenolics identified by UPLC-QTOF-MS.

Only 10 phenolic compounds were present in the entire study, at least in one treatment, as part of the oil profile, water, or eggplant samples (Table 1 and Table 3). Figure 1 shows the three most abundant phenolic compounds quantified in the samples. Gallic acid, pinoresinol, o-coumaric, p-hydroxybenzoic and p-hydroxyphenylacetic acids, oleuropein, rutin, hydroxytyrosol, and luteolin were not detected.

After thermal treatment, trans-caffeic and sinapic acids were added to the AVO profile to sum up, nine compounds (Table 1). Some authors [3] reported trans-caffeic and sinapic acids in AO together with other phenolics not detected in our study, as gallic, 3,4-dihydroxyphenylacetic, and p-hydroxybenzoic acids. Deep frying is the technique that most enriched the profile of the AVO. Otherwise, W/O boiling impoverished the profile in a way that just remained in detectable amounts the tyrosol, p-vanillin, and quercetin. The content of phenolics already present in the raw AVO increased after being used as frying media, in the case of tyrosol (314%), p-vanillin (279%), and vanillic acid (20%), but remained steady trans-caffeic acid, ferulic acid, chlorogenic acid, apigenin, and quercetin. Including oil during vegetable boiling led to the depletion of ferulic acid and apigenin and reduced tyrosol (−910.70%) and quercetin (−810.90%) contents the concentrations of p-vanillin and chlorogenic acid did not show statistical differences from the raw AVO. Tyrosol was the compound that most accounted for TPC in fresh and processed AVO; however, its amounts never reached the fixed value (250 mg/kg) to bear the health claim about protection of low-density lipoprotein particles from oxidative damage for the general population as is stated for olive oil [66].

In the phenolic profile of raw eggplant, only were detected chlorogenic acid, tyrosol, and p-vanillin, being the first predominant; nevertheless, all cooking techniques enriched the vegetable with new phenols summing up a maximum of eight different compounds (Table 1). After all culinary treatments, it was added trans-caffeic acid to the vegetable phenolic profile. In addition, deep-frying also contributed to p-coumaric acid, ferulic acid, sinapic acid, and quercetin. The phenols added were reported before in the fresh or processed eggplant without oil, as in the case of chlorogenic acid, caffeic acid, and vanillin in fresh and microwaved eggplant [61]; also as in the case of ferulic and p-coumaric acids [24,62]. The concentration of individual phenols found in the fresh vegetable phenolic profile increased with all the culinary techniques tested standing out deep-frying. The 1196% increment in chlorogenic acid concentrations most explained the rise in TPC in eggplant after cooking, although the concentrations of p-vanillin and tyrosol raised, respectively, by 4985% and 1796% after frying the vegetable; this correlation between chlorogenic acid and TPC was previously stated [67].

Water gained phenols after bot boiling treatments. Raw foods contributed to the water with tyrosol, p-vanillin, and trans-caffeic and chlorogenic acids. The measured chlorogenic acid concentrations in the recovered water were lower when AVO was used, but the other phenols did not vary with the boiling modality used.

Our results in deep-fried AVO did not agree with the multiple reports of net decreases in phenol concentration that ranges from 40 to 80% after heat treatment with or without food [68,69]. But, coincidences with other oil reports showed mixed tendencies in the phenols depending on the compound analyzed and the vegetable processed. For example, during the roasting of vegetables with extra virgin olive oil (EVOO), the detrimental effects on the oil predominated over the stability of the phenols, and just hydroxytyrosol acetate increased in concentration [70]. To date, few studies in vegetable oils consistently report the incorporation of new phenolics into the profile or increments of the already existing after processing. Studies of sautéing and deep-frying of vegetables in EVOO under realistic conditions have referred to the incorporation of chlorogenic acid into the oil; and the increase in concentrations of the already present gallic, dihydroxybenzoic, hydroxybenzoic, vanillic, and hydroxiphenilacetic acids, as well as in luteolin, and apigenin [28]. Also, polyphenols were detected from tomato, garlic, and onion, such as ferulic acid, naringenin, and quercetin in the used olive oil after sofrito sauce elaboration [31]. To date, the incorporation of new compounds and increases in the concentrations of the already present is explained by the fact that solubility and dispersal in oils increase with high-temperature conditions, although these compounds exhibit poor solubility in oils [71]. The phenols reduction in AVO—used for W/O boiling—is clearly explained by the leaching of the oil into the water. The water-soluble nature of polyphenols drives the movement; this phenomenon was referred during the cooking of potatoes, carrots, and onions in the presence of olive oil [29,72].

Concerning eggplant, the tendencies to the increments in the number of phenolic compounds present and its concentrations after frying and boiling eggplant agreed with many studies [8,10,24]. The increment observed has been mainly explained by the food matrix softening effect of thermal treatment and the hydrolysis and isomerization reactions of those phenols covalently or ionically linked to macromolecules such as polysaccharides or proteins of the cell-wall structure of eggplant [16,58]. This phenomenon depends on the time of processing, temperature, and food geometry, among other factors, in such a way that longer times and higher temperatures diminishes phenol concentrations [58,68]. However, the food matrix also influences the release and stability [10] and also depends on the compound analyzed. It has been seen an increase of 74% in total hydroxycinnamic acids after frying treatment, but a decrease of 27% after boiling eggplant; this increment was attributed to the parallel increase of the free forms di-caffeoyl-quinic acids and hydroxycinnamic acid-amides [24]. Also have been reported increases in chlorogenic acid content in eggplant after thermal treatment such as frying in EVOO [10], or grilling, roasting, and baking [23]; or after wet cooking procedures as steaming, pressure cooking, and boiling [73]. Massive trans-esterification of 5-O-caffeoylquinic acid to 3-O-caffeoylquinic has been referred to as the mechanism underlying chlorogenic acid increase due to high temperature; this effect was also reported in artichoke [74]. In our study, quercetin was not found in raw vegetable but was detected only after frying; it has been reported that quercetin can result from deglycosylation of quercetin-3-O-glycoside after thermal treatment [75]. In our experiments, caffeic acid was incorporated into the phenolic profile after frying, boiling, and W/O boiling; in contrast, the phenolic acids, p-coumaric, ferulic, and sinapic were incorporated only after frying. The mentioned phenolics have increased in spinach during blanching and boiling processes [76]; but, has been measured a decline of caffeic, sinapic, and ferulic acids after conventional cooking of broccoli [77]. In vegetable based juices were seen chlorogenic acids consisting in esters formed between quinic acid and trans-cinnamic acids (caffeic, ferulic, or p-coumaric acids) [78]. During roasting of coffee was reported instability of this compounds at high temperature processing [79].

#### 3.2.2. Lipidic Fraction

##### Fatty Acid Profile

Percentages of the oleic, palmitic, and linoleic fatty acids remained steady between treatments; there were no significant differences (*p* ≥ 0.05) between the raw or used oil and the lipidic fractions obtained from eggplant after treatments (Table 2). However, in AVO, differences were significant in the proportions of FAs with three unsaturations, going to reduction until exhaustion after W/O boiling, and DGLA did it after deep frying. The behavior of major fatty acids has been seen before during the roasting of carrots, onions, and potatoes with EVOO; in this case, all the oil samples investigated fully preserved the level of fatty acid unsaturation [70].

The fatty acid profile of vegetable oils influences its physical properties during frying, especially levels of viscosity and heat transfer properties [20]. Its degree of unsaturation, however, modifies the stability during thermal treatment [21]. It is well known that high oleic oils such as AO show high stability during heat treatments such as cooking. Also, it has been demonstrated that monounsaturated acids are associated with higher specific heat values in these oils, opposite to oils with higher content of polyunsaturated acids like virgin flaxseed oil; the presence of polyunsaturated fatty acids reduces the specific heat values leading to lower stability during thermal treatment [21]. The oil used for frying plays an essential role in the improvement of the fat quality of foods. It has been mentioned that the choice of the oil is entirely reflected in the final product, as was seen during low-fat frying processing of potato with sunflower, soybean, canola, and olive oil [27], or as recognized from the use of olive oil, which enriches the food with healthy monounsaturated fatty acids from the oil during frying [80]. The same consideration can be done with avocado oil since its fat profile includes more than 60% of monounsaturated fatty acids and can be extended for W/O boiling or all the cooking techniques that include oil during processing. From the above mentioned, non-fatty foods, like vegetables, are excellent carriers of healthy fats since their amount of oil after processing comes exclusively from that absorbed from the cooking media.

##### Total and Individual Sterol Stanol Contents

Total sterol content ranged from 4030.1 mg/kg in the oil added in W/O boiling and 3557.8 mg/kg in the lipidic fraction from W/O boiled eggplant (Table 2). AVO from the three treatments attained the highest contents, and its media remained grouped without significant differences (*p* ≥ 0.05). The lipidic fraction from the eggplant had the lowest TSC without differences between fried and W/O boiled samples (*p* ≥ 0.05).

The proportion of major sterols (β-sitosterol, campesterol, and Δ5-avenasterol) in the lipidic fraction of eggplant or the AVO recovered from the culinary assays remained unchanged among treatments, except that of campesterol in the fat fraction recovered from W/O boiled eggplant. The fat extracted from cooked eggplant showed significant changes (*p* ≤ 0.05) in sterol profile whit respect to fresh oil. W/O boiling increased campesterol and stigmasterol percentages; but lowered methilcholesterol, Δ5- and Δ7-avenasterol content. After deep-frying, occurred a rise in the campesterol and stigmasterol proportions and a lowering of Δ7-avenasterol. The percentages of the rest sterols analyzed in the aubergine lipidic fractions remained grouped with the original values of raw oil used during cooking. About stanols, only Δ7-stigmastenol percentage resulted in a significant difference, showing an increment after W/O boiling, and a rise following deep frying.

In our study, the percentages of individual sterols in the AVO used as coking media showed statistically significant differences due to the deep-frying processing. It was measured a lowering in Δ7-avenasterol and the depletion of brassicasterol. Deep-frying changed stanols in a way that diminished Δ7-stigmastenol proportion but augmented that of Δ5, 24-stigmastadienol. W/O boiling did not affect the sterol/stanol profile of AVO.

The most frequent studies are those referring to the reduction in sterol content in oils after thermal treatments. In avocado oil, heating of 180 °C for 3 h reduced the TSC from 3.4 to 2.4 mg/g [22]. In rapeseed oil, a 48 h heating (170 °C) decreased total sterol content (21.2 to 46.1%); and the contents of brassicasterol (7.1 to 44.4%), campesterol (13.8 to 53.2%), stigmasterol (33.3 to 50%), β-sitosterol (14.7 to 43.6%), and avenasterol (34.4 to 76.9%) [19]. Also has been demonstrated in rapeseed oil that sterol contents lower as the level of processing increases (crude, refining, and partial hydrogenation). Additionally, it was demonstrated that increments in heating time and temperature increased the loss rate of soybean germ phytosterols in different lipid matrix, including soybean germ oil, olive oil, and lard matrix [30]. Although those reductions were found in simulations without foods, using longer times than those used in our study, they already corresponded with the tendencies measured in AVO by us. It is also confirmed from the results observed in oils used in potato processing through pan- and deep-frying, where sterol content decreased 1.7% for cottonseed oil, 11.9% for sunflower oil, 8.5% for palm oil, and 2.6% for virgin olive oil [7].

Oxidation reactions are the leading factor mediating the phytosterols loss in oil. On the initial phases of heating processing, the present tocopherols slow down sterol oxidation; but once exhausted, the oxidation products rise according to the oil studied and the proportion of the sterol species [81]. During the heating of pressed oils, for example, the case of pressed rapeseed oil has been observed a sharp increase of 5α,6α-epoxy, and 5β,6β-epoxy derivatives of β-sitosterol after 48 h. Products from β-sitosterol accounted for a significant part, but campesterol and stigmasterol oxidation products also were present in a lower amount. The dominant campesterol oxidation products are 5β,6β-epoxy, 7-ketocampesterol; while the main groups of stigmasterol oxidation products are 7α and 7β-hydroxy derivatives [19]. Consequently, the differentiated degree of depletion of sterols accounted for the rearrangement in the sterol profiles reported by us as significant changes in the analyzed fractions.

In contrast, in food matrixes has been reported increases in SC during the cooking process. In potatoes, the rise came from the absorption of frying oil when processed through pan-frying (175 °C, 6 min) or deep-frying (170 °C, 9 min) in sunflower, palm, cottonseed, virgin and olive oils [7]. It was observed that phytosterols are distributed almost uniformly between frying oil and fried potatoes being β-sitosterol the predominant followed by campesterol, Δ5-avenasterol, and stigmasterol; as a result, their concentrations in potatoes could be estimated by determining their content in the frying oil taking into account the mass of absorbed oil [7]. The rise in TSC observed in our study may be mainly attributed to the stability of the compounds previously referred, and the concentration effect from moisture reduction after thermal treatments of eggplant, instead of to the release of compounds bonded to the cellular structures, since eggplant has been referred as a very poor sterol source [64].

### 3.3. Multivariate Analysis

General discriminant analysis (GDA) provided different discriminant accuracy for each data set. In all cases, forward stepwise GDA made it possible to select the discriminating parameters and reduce the number of variables.

**Figure 2:**
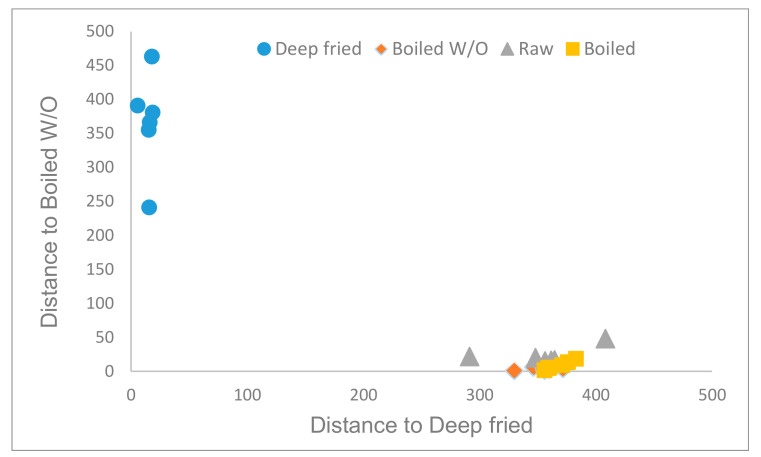
Cooman′s graph of the Mahalanobis distances for samples′ classification according to the culinary techniques using the hydrophilic compounds.

GDA of the hydrophilic fraction considering only the culinary treatment retained 2 parameters (fat and ABTS) and 6 phenolic compounds (chlorogenic, ferulic, p-coumaric, and sinapic acids; tyrosol and quercetin). Mahalanobis distances between the culinary treatments were highly statistically significant (*p* < 0.01), except for the distance between boiled and boiled W/O (*p* = 0.05). The obtained model made it possible to classify correctly 88.9% of samples according to the culinary treatment (Figure 2). Misclassification was observed for boiled (83.3% correctly classified) and boiled W/O (77.8% correctly classified) samples since there was confusion between these two techniques. Confusion among these culinary methods could be explained since the addition of AVO during boiling does not apport enough phenolic compounds due to their low concentration in the oil.

**Figure 3:**
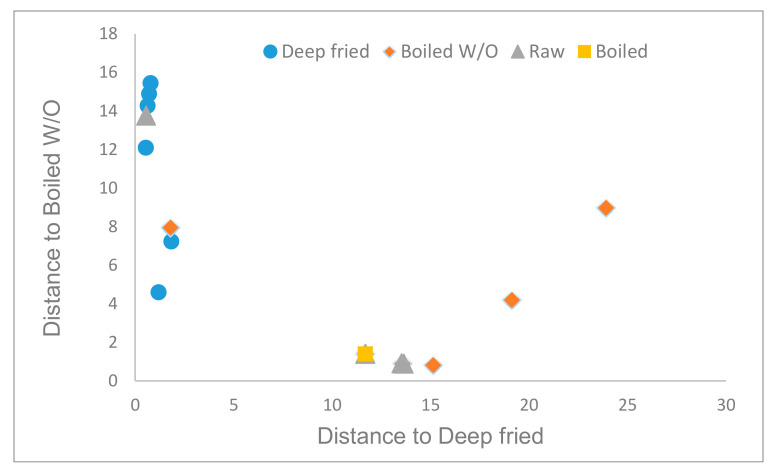
Cooman′s graph of the Mahalanobis distances for samples′ classification according to the culinary techniques using the lipidic compounds.

On the other hand, GDA of the lipidic fraction considering only the culinary treatment retained only one fatty acid (cis dihomo-γ-linolenic acid) and one sterol (stigmasterol). Mahalanobis distances between deep-fried and the other techniques were highly statistically significant (*p* < 0.01). No significant distances were observed for the other techniques. The obtained model made it possible to classify correctly only 62.9% of samples according to the culinary technic (Figure 3). In the case of deep-fried and boiled, 100% of samples were correctly classified. Misclassification was observed for boiled W/O samples (55.6% correctly classified) and raw samples (0% correctly classified). Boiled W/O samples were confounded with deep-fried and raw, while raw samples were confounded with the other three techniques. Confusion among these culinary methods could be explained since AVO apports the total lipidic compounds.

**Figure 4:**
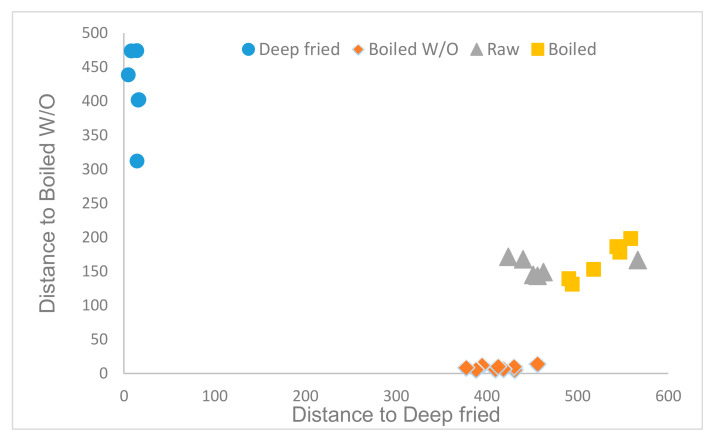
Cooman′s graph of the Mahalanobis distances for samples′ classification according to the culinary techniques using the hydrophilic and lipidic compounds.

GDA of the data including hydrophilic and lipidic fractions as one unique dataset and considering only the culinary treatment made it possible to retain 2 parameters (total phenol and DPPH), 6 phenolic compounds (p-vanillin, tyrosol, quercetin; p-coumaric, chlorogenic, and ferulic acids), one fatty acid (linoleic acid), and one stanol (Δ5, 24-stigmastadienol). Mahalanobis distances were highly statistically significant (*p* < 0.01) for all the treatments. The obtained statistical model made it possible to classify 100% of samples (Figure 4) correctly.

As seen in the GDA of the whole data set, better discrimination between culinary technics is obtained using compounds from both fractions instead of separately. From the analysis, we observed that each culinary method assayed leads to a specific compositional profile that includes both hydrophilic and lipidic fractions, independently of the foodstuff.

**Figure 5:**
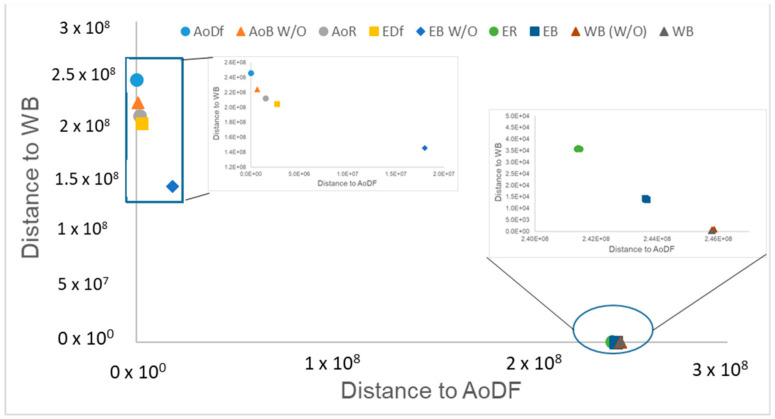
Cooman′s graph of the Mahalanobis distances for samples′ classification according to the food and the culinary techniques using hydrophilic and lipidic compounds. AoDf: Avocado oil-Deep frying, AoB W/O: Avocado oil-Boil W/O, AoR: Avocado oil-Raw, EDf: Eggplant-Deep frying, EB W/O: Eggplant-Boil W/O, ER: Eggplant-Raw, EB: Eggplant-Boil, WB (W/O): Water-Boil W/O, and WB: Water-Boil.

Finally, GDA of the whole dataset considering the combination between the foods and the culinary treatments made it possible to select four parameters (fat, dry matter, total phenol, and DPPH), five phenolic compounds (chlorogenic and ferulic acids; tyrosol, p-vanillin, and quercetin), five sterols (campesterol, stigmasterol, β-sitosterol, Δ7-campesterol, and Δ5-avenasterol), and two fatty acids (palmitoleic and linolenic acids). Mahalanobis distances were highly statistically significant (*p* < 0.01) for most of the treatments, except for the distance between WB (O/W) and WB (*p* = 0.03). The obtained statistical model made it possible to classify correctly 100% of samples (Figure 5).

From the results of the final GDA, it was demonstrated that the final chemical profile of the foods is the result of the movement of hydrophilic and lipophilic fractions between them. This movement created characteristic profiles for each combination of food and culinary treatment.

## 4. Conclusions

From the results of the final GDA, it was demonstrated that the final chemical profile of the foods is the result of the movement of hydrophilic and lipophilic fractions between them. This movement created characteristic profiles for each combination of food and culinary treatment. In this study, AVO was the only contributor of lipophilic functional compounds, leading to the enrichment of the processed eggplant. However, eggplant was the main contributor of the hydrophilic fraction to the AVO and water. During deep-frying, it was demonstrated that an active migration of the high biological value lipophilic fraction was found in AVO to eggplant. This migration improved the functional properties of eggplant by adding sterols and unsaturated fatty acids to the naturally occurring polyphenols. When the eggplant was processed by boiling, the addition of AVO brings the benefit of the inclusion of high biological lipophilic compounds to the food preparation without an excessive increase of its caloric content. Consuming the boiling water made it possible to profit from the phenolic compounds lixiviated from the vegetable and the AVO.

## Figures and Tables

**Table 1:** Analysis of variance and Fisher′s LSD test of composition and hydrophilic compounds of avocado oil, eggplant and processed water according to four culinary treatments *.

Compounds	Avocado Oil		Eggplant					Water	
	Raw	Deep-Fried	Boiled W/O	Raw	Deep-Fried	Boiled W/O	Boiled	Boiled W/O	Boiled
Composition ^†^									
Moisture	0.1 ^a^	0.05 ^a^	0.2 ^a^	93.3 ^d^	45.9 ^b^	89.6 ^c^	96.1 ^e^	99.4 ^f^	99.5 ^f^
Dry Matter	0.0 ^a^	0.0 ^a^	0.0 ^a^	6.7 ^c^	54.1 ^e^	11.8 ^d^	3.5 ^b^	0.6 ^a^	0.5 ^a^
Fat	99.9 ^d^	99.9 ^d^	99.8 ^d^	0.1 ^a^	37.8 ^c^	6.4 ^b^	0.02 ^a^	0.0 ^a^	0.0 ^a^
Phenolic compounds ^‡^									
Tyrosol	33.5 ^b^	138.8 ^d^	3.1 ^a^	3.7 ^a^	65.8 ^c^	4.2 ^a^	2.8 ^a^	2.5 ^a^	1.5 ^a^
P-vanillin	14.6 ^c^	3.8 ^b^	4.4 ^b^	3.5 ^b^	0.54	2.5 ^ab^	27.0 ^d^	0.7 ^a^	0.7 ^a^
P-Coumaric	0.0 ^a^	0.0 ^a^	0.0 ^a^	0.0 ^a^	3.0 ^b^	0.0 ^a^	0.0 ^a^	0.0 ^a^	0.0 ^a^
Vanillic Acid	1.3 ^b^	1.5 ^b^	0.0 ^a^	0.0 ^a^	0.0 ^a^	0.0 ^a^	0.0 ^a^	0.0 ^a^	0.0 ^a^
Trans Caffeic Acid	0.0 ^a^	0.4 ^ab^	0.0 ^a^	0.0 ^a^	7.5 ^c^	0.4 ^b^	0.5 ^b^	0.3 ^ab^	0.4 ^ab^
Ferulic Acid	1.4 ^b^	1.1 ^b^	0.0 ^a^	0.0 ^a^	18.0	0.0 ^a^	0.0	0.0 ^a^	0.0 ^a^
Sinapic Acid	0.0 ^a^	0.5 ^b^	0.0 ^a^	0.0 ^a^	3.8 ^c^	0.0 ^a^	0.0 ^a^	0.0 ^a^	0.0 ^a^
Apigenin	0.4 ^b^	0.3 ^b^	0.0 ^a^	0.0 ^a^	0.0 ^a^	0.0 ^a^	0.0 ^a^	0.0 ^a^	0.0 ^a^
Quercetin	1.9 ^c^	1.9 ^c^	0.4 ^b^	0.0 ^a^	1.8 ^c^	0.0 ^a^	0.0 ^a^	0.0 ^a^	0.0 ^a^
Chlorogenic Acid	0.3 ^a^	5.6 ^a^	4.5 ^a^	37.8 ^b^	451.4 ^f^	90.0 ^c^	126.5 ^d^	115.6 ^cd^	167.5 ^e^
Total Phenolics ^#^	8.9 ^a^	8.5 ^a^	8.9 ^a^	35.3 ^b^	213.4 ^e^	40.3 ^b^	37.2 ^b^	186.9 ^d^	131.8 ^c^
Antioxidant activiy ^&^									
DPPH	0.4 ^ab^	0.1 ^a^	0.1 ^a^	0.9 ^c^	8.8 ^f^	1.9 ^e^	1.5 ^d^	0.7 ^bc^	0.3 ^ab^
ABTS	1.7 ^b^	1.3 ^a^	1.7 ^b^	2.1 ^c^	12.2 ^f^	3.7 ^e^	2.4 ^d^	1.6 ^b^	1.3 ^a^

^†^ Expresed as %; ^‡^ mg/kg f.w.; ^#^ mg GAE/kg f.w.; ^&^ μmol TE/g f.w. * Raws with different letter show statistically significant differences (*p* < 0.05).

**Table 2:** Analysis of variance and Fisher′s LSD test of the lipidic fraction of avocado oil and eggplant according to three culinary treatments *.

Compound	Avocado Oil			Eggplant	
	Deep-Fried	Boiled W/O	Raw	Deep-Fried	Boiled W/O
Sterols and stanols ^†^					
Brassicasterol	0.0 ^a^	0.01 ^b^	0.01 ^b^	0.01 ^b^	0.01 ^b^
Methilcholesterol	0.7 ^b^	0.7 ^b^	0.8 ^b^	0.7 ^b^	0.7 ^a^
Campesterol	6.0 ^a^	5.9 ^a^	5.9 ^a^	6.1 ^b^	6.4 ^c^
Campestanol	0.02 ^a^	0.02 ^a^	0.03 ^a,b^	0.02 ^a^	0.04 ^b^
Stigmasterol	0.3 ^a^	0.4 ^a^	0.4 ^a^	1.8 ^b^	3.5 ^c^
Δ7-Campesterol	0.04 ^b^	0.03 ^a^	0.03 ^a^	0.03 ^a,b^	0.03 ^ab^
Δ5,23-Stigmastadienol	0.4 ^c^	0.3 ^a,b^	0.3 ^b,c^	0.3 ^a,b^	0.2 ^a^
Clerosterol	1.5 ^a^	1.5 ^c^	1.5 ^ab^	1.5 ^a^	1.5 ^a,b^
β-sitosterol	85.2 ^a^	85.1 ^a^	85.2 ^a^	83.9 ^a^	84.5 ^a^
Sitostanol	0.3 ^a^	0.4 ^a^	0.4 ^a,b^	0.4 ^b,c^	0.4 ^c^
Δ5-Avenasterol	4.9 ^b^	5.1 ^b^	5.1 ^b^	4.8 ^b^	2.2 ^a^
Δ5,24-Stigmastadienol	0.3 ^c^	0.2 ^b^	0.2 ^b^	0.2 ^a^	0.2 ^ab^
Δ7-Stigmastenol	0.05 ^a^	0.05 ^b^	0.06 ^c^	0.05 ^b^	0.07 ^d^
Δ7-Avenasterol	0.12 ^a^	0.1 ^c^	0.1 ^c^	0.1 ^b^	0.1 ^a^
Total sterols ^‡^	3890.6 ^b^	4030.1 ^b^	3881.5 ^b^	3671.7 ^a^	3557.8 ^a^
Fatty acids ^#^					
Palmitic	13.6 ^a^	15.6 ^b^	14.3 ^a^	14.1 ^a^	14.6 ^a,b^
Palmitoleic	5.7 ^a^	6.8 ^c^	6.4 ^b,c^	6.2 ^a,b^	5.9 ^a,b^
Oleic	65.9 ^a^	63.5 ^a^	65.3 ^a^	64.1 ^a^	64.7 ^a^
Linoleic	9.4 ^a,b^	9.7 ^b^	9.1 ^ab^	9.0 ^a,b^	8.6 ^a^
Linolenic	0.6 ^b,c^	0.0 ^a^	0.4 ^b^	0.7 ^c^	0.6 ^c^
Gondoic	1.0 ^b^	0.0 ^a^	0.3 ^a^	1.1 ^b^	0.9 ^b^
Dihomo-γ-linolenic	0.0 ^a^	0.0 ^a^	0.1 ^a^	0.6 ^b^	0.6 ^b^
Vaccenic	3.9 ^a^	4.4 ^a^	4.0 ^a^	4.2 ^a^	4.0 ^a^

^†^ Expressed as %; ^‡^ mg/kg f.w.; ^#^ Expresed as %; * Raws with different letters show statistically significant differences (*p* < 0.05).

**Table 3:** Individual phenolic compounds identified by UPLC-QTOF-MS.

Phenolic Compound	Name	Molecular Formula	[M-H]^−^ Calculated	[M-H]^−^ Experimental	R_t_ (min)	MS Fragments	Linear Equation (Quantification)	R^2^
* 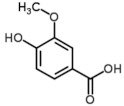 *	Vanillic acid	C_8_H_8_O_4_	167.0344	167.0351	3.97	151.62 107.52 90.44 79.43	y = 12263x + 133147	0.980
* 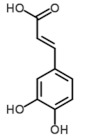 *	Caffeic acid	C_9_H_8_O_4_	179.0344	179.0336	4.21	96.35 58.28 118.48 78.27	y = 1148.45x + 485.875	0.998
* 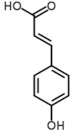 *	p-coumaric acid	C_9_H_8_O_3_	163.0395	163.0401	4.81	118.35 92.42 96.36 42.21	y = 1248.29x + 8339.28	0.992
* 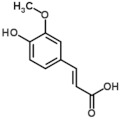 *	Ferulic acid	C_10_H_10_O_4_	193.0501	193.0139	3.90	133.51 177.64 58.28 116.43	y = 8583.45x + 61398	0.993
* 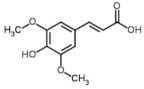 *	Sinapic acid	C_11_H_12_O_5_	223.0606	223.0584	3.91	163.25 207.79 192.71 148.60	y = 7747.82x + 28708.3	0.995
* 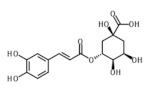 *	Chlorogenic acid	C_16_H_18_O_9_	353.0873	353.1018	2.41	190.74 84.40 92.39 96.32	y = 17881.6x + 72017.9	0.998
* 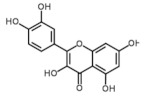 *	Quercetin	C_15_H_10_O_7_	301.0348	301.0355	5.52	150.63 178.67 106.47 120.54	y = 15094.4x + 61272.8	0.985
* 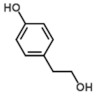 *	Tyrosol	C_8_H_10_O_2_	137.0603	137.0531	5.47	105.63 105.72 80.35 58.27	y = 6835.41x −29.087	0.999
